# Large-scale multiethnic electronic health record resource for diabetes complications research: the North West London Diabetes Cohort (NWLDC) – cohort profile

**DOI:** 10.1136/bmjopen-2025-114566

**Published:** 2026-09-04

**Authors:** Zeinab Schaefer, Schanhave Santhirasekaran, Alasdair Warwick, Tabitha Grainger, Aroon Hingorani, Adnan Tufail, Luke Dixon, John Anderson, Dimitrios Moutzouris, Alicja R Rudnicka, Iain N Roy, Christopher G Owen

**Affiliations:** 1 Department of Global, Public and Population Health and Policy, City St George’s University of London, London, UK; 2 University College London Institute of Cardiovascular Science, London, UK; 3 NIHR Biomedical Research Centre at Moorfields Eye Hospital NHS Foundation Trust and UCL Institute of Ophthalmology, London, UK; 4 Cardiovascular and Genomics Institute, City St George’s University of London, London, UK; 5 Department of Surgery and Cancer, Imperial College London - Hammersmith Campus, London, UK; 6 Diabetes and Endocrinolgy, Homerton University Hospital, London, UK; 7 Barts and The London School of Medicine and Dentistry Blizard Institute, London, UK; 8 Guy’s and St Thomas’ NHS Foundation Trust, London, UK

**Keywords:** EPIDEMIOLOGY, Electronic Health Records, DIABETES & ENDOCRINOLOGY, Observational Study

## Abstract

**Abstract:**

**Purpose:**

The North West London Diabetes Cohort is established to provide systematic characterisation of a large diabetes population as a foundation for complications research and prognostic modelling. Many predictive modelling studies neglect the essential descriptive characterisation of underlying cohorts, focusing narrowly on model accuracy. This cohort profile addresses this gap by comprehensively describing the demographic composition, clinical characteristics and complication incidence patterns. The notably diverse, multiethnic population enables examination of ethnic disparities and supports future development of reliable prognostic models and evidence-based prevention strategies for diabetes complications.

**Participants:**

At baseline, 337 271 patients with diabetes were identified. It includes 279 067 patients with type 2 diabetes, 17 638 with type 1 diabetes, 33 590 with gestational diabetes and 6916 with unspecified diabetes. The earliest diabetes diagnosis dates to January 1932, with data updated to 27 May 2025.

**Findings to date:**

This cohort profile describes baseline characteristics of patients with comprehensive data collected on demographics (age, sex, Deprivation Index, ethnicity), clinical measures (glycated haemoglobin, body mass index, blood pressure, lipids and estimated glomerular filtration rate) and 14 major diabetes complications tracked longitudinally. Key findings for patients with type 2 diabetes reveal diabetic retinopathy as the most common complication (74.6 per 1000 person-years), followed by hypertension (51.0) and kidney disease (31.4). Cumulative incidence analyses using the Aalen-Johansen estimator, which accounts for mortality as a competing risk, demonstrated significant ethnic disparities, with black, Asian, mixed and other ethnic groups showing elevated risk compared with white patients. Time-varying Cox models identified strong clustering between cardiovascular and renal complications, confirming a cardiometabolic-renal syndrome. Mental health conditions (depression and anxiety) were prevalent throughout the disease timeline, occurring both before and after diabetes diagnosis.

**Future plans:**

This cohort will be used as a platform for developing and validating prognostic models for diabetes complications, enabling risk stratification and targeted interventions. Future work will incorporate medication data to refine diabetes type classification, examine the effectiveness of antidiabetic medications in preventing different complications and address demographic differences in prognostic model performance and prediction accuracy. To better characterise lifestyle, further interrogation of electronic health record data will examine recording of advice given, including dietary advice, referral to weight management schemes and presence of alcohol consumption codes.

**STRENGTHS AND LIMITATIONS OF THIS STUDY**

- North West London Diabetes Cohort comprises 337 211 patients with linked data across primary, secondary, mental health and social care, representing one of the largest multiethnic diabetes cohorts in the UK.
- Substantial representation from Asian and black populations, groups often under-represented in research but disproportionately affected by diabetes.
- Richness of longitudinal measures including clinical data, laboratory tests, complications and socioeconomic indicators that enable comprehensive multidomain analyses.
- Misclassification of diabetes type remains a challenge due to overlaps in Systematised Nomenclature of Medicine – Clinical Terms (SNOMED CT) coding and disease evolution over time; while hierarchical classification rules were applied, residual misclassification may persist.
- Cumulative incidence estimates assume independent censoring; patients lost to follow-up may differ in risk, which could lead to underestimation or overestimation.
- Absence of medication data in the current analysis limited refinement of diabetes type classification, though future work will incorporate prescriptions.

## Introduction

### Why was the cohort set up?

 Diabetes mellitus affects 589 million adults worldwide, with forecasts suggesting that prevalence will reach 853 million by 2050.^1^ Since 2010, there has been a worrying increase in type 2 diabetes mellitus (T2DM) among young adults.^2^ This earlier onset lengthens the period of exposure to disease and increases cumulative risks of complications, which include cardiovascular disease, neuropathy, nephropathy and retinopathy.^3–5^ Within the UK, the burden is particularly heavy, with National Health Service costs associated with diabetes reaching £14 billion in 2021/22, nearly 60% of which were due to complications.^6^

The prevention or mitigation of complications offers the greatest opportunity to improve patient outcomes and reduce healthcare costs. Reliable predictive models are essential to anticipate complications and intervene effectively. Yet prediction requires an in-depth understanding of the patient population, including clinical heterogeneity, baseline risk factors and the quality of electronic health data. Many existing diabetes prediction studies focus narrowly on model accuracy, neglecting the foundational characterisation of the underlying cohorts that is critical for meaningful modelling.^7^

Increasingly, ‘real world’ electronic health record data are being used, given large-scale routine collection. However, these studies face challenges such as accurate case identification (with date of disease diagnosis as opposed to onset), data quality assessment and handling of missing information.^8 9 10^ Systematic cohort characterisation is an important prerequisite for predictive research. With the rise of secure integrated health data platforms, opportunities to use real-world evidence for diabetes research have grown. A leading example is the OneLondon Health Data Strategy, delivered through the OneLondon programme.^11^ This initiative unifies data across London’s five Integrated Care Systems: North Central London, North East London, South East London, South West London and North West London (NWL).

A cornerstone of this programme is the NWL Whole Systems Integrated Care (WSIC) database; this is a linked, longitudinal dataset developed by NWL Integrated Care Board. The de-identified WSIC database is used for this research study.^12^ The WSIC database links primary care, acute care, mental health, community care and social care data for over 5 million patients to provide a comprehensive linked, longitudinal dataset. This comprehensive coverage provides an unparalleled opportunity to examine diabetes progression, identify complications and lay the foundation for risk stratification research.

Against this background, the NWL Diabetes Cohort (NWLDC) was established to provide a robust descriptive foundation, characterising patients by demographics, clinical measures and data quality, and to enable predictive modelling research into complications and outcomes.

## Cohort description

### Study population

The NWLDC within the WSIC database covers 5 062 171 patients as of 27 May 2025 comprising 2 882 713 actively registered patients, 1 914 283 previously registered and 265 175 deceased. The earliest diagnosis dates were January 1932, 1942, 1939 and 1942 for T1DM, T2DM, gestational diabetes mellitus (GDM) and other diabetes types, respectively. General practice records are coded using Systematised Nomenclature of Medicine – Clinical Terms (SNOMED CT), which includes over 350 000 numerical codes covering diagnoses, symptoms, procedures and other health information.

Using a curated list of diabetes SNOMED CT codes, informed by the Quality and Outcomes Framework diabetes definitions, we identified 337 211 patients with diabetes-related codes. Patients were classified into four categories: T2DM (n=279 067), T1DM (n=17 638), GDM (n=33 590) and unspecified/other types (n=6916) (online supplemental figure S1).

During classification, overlaps emerged. For example, 11 213 patients were coded as both T1DM and T2DM, and 6143 as both GDM and T2DM. To resolve this, diagnosis dates were set to the earliest registration, but diabetes type was determined by the latest classification. For instance, patients first labelled with T2DM but later correctly reclassified as T1DM were assigned a final T1DM status. Similarly, overlaps between GDM and T2DM were resolved by prioritising the earliest recorded type, recognising the known progression from GDM to T2DM. Remaining unclassifiable patients were placed in the unspecified category. The effect of these adjustments on the age distribution for T1DM and GDM is shown in online supplemental figures S2 and S3. After corrections, 337 211 unique patients with diabetes remained, forming the core cohort for analyses.

Diabetes complications were identified using the same code-based approach. For each of 14 major complications, a curated SNOMED CT code list was assembled and reviewed for clinical validity, and a patient was considered affected if any qualifying code appeared in their record, with the earliest such code taken as the date of onset for timing relative to diabetes diagnosis. Because complications are determined from clinician-entered diagnostic codes rather than measurement thresholds, their identification is unaffected by changes in diagnostic cut-offs over the study period (ie, evolving blood pressure thresholds for hypertension). The 14 complications were retinopathy, hypertension, kidney disease, coronary heart disease, depression, anxiety disorders, heart failure, stroke, dementia, peripheral artery disease, foot ulcer, transient ischaemic attack, liver disease and amputation (major and minor), with full SNOMED CT codelists provided as online supplemental material. Kidney disease is a composite outcome, comprising chronic kidney disease stages 1–2 and stages 3–5, diabetic nephropathy and renal dialysis.

### How often have they been followed up?

The WSIC database is continuously updated, integrating data from multiple care settings. Patients contribute data throughout their lifetime of registration in NWL general practices, across general practitioner, hospital, mental health and social care records. For the current analyses, however, all work was performed on a static snapshot up to 27 May 2025. This approach ensured reproducibility and consistency, given the dynamic nature of the database.

**Figure 1:**
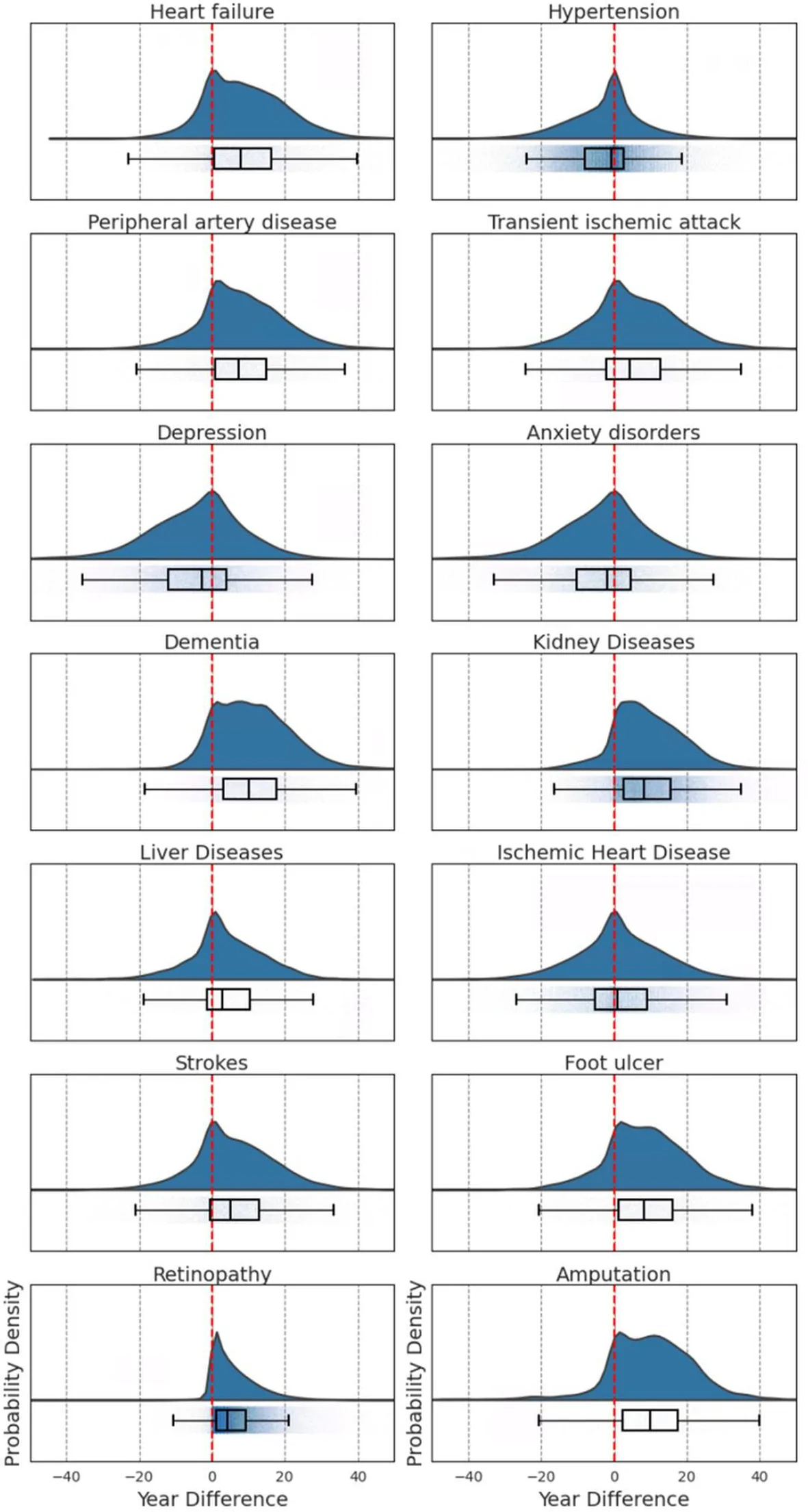
Density plots show when 14 major medical conditions first occur within 30 years before or after diabetes diagnosis (year 0). Negative values indicate pre-diabetes occurrence; positive values show years since diabetes onset.

There is heterogeneity in the amount of data available for an individual depending on patient registration duration and survival. Some patients have data stretching back decades, while others contribute shorter histories. Complications are tracked longitudinally, with temporal analyses estimating incidence relative to the date of diabetes diagnosis. Figure 1 shows temporal patterns of 14 major complications of diabetes, including, cardiovascular disease and mental health disorders. Mental health disorders such as depression and anxiety show wide distributions around diagnosis, while severe events such as amputations, associated with peripheral arterial disease, occurred at variable times after diabetes diagnosis, but were on average a decade after. This enables time-to-event analyses and long-term follow-up patterns to be studied robustly.

### What has been measured? (variables)

A broad range of demographic, clinical and socioeconomic measures are available in WSIC. Key demographic features include age at diagnosis, sex, ethnicity and socioeconomic deprivation deciles. Ethnicity is a major strength of this dataset, with diverse representation across Asian, black, mixed, white and other ethnic groups. Sociodemographic and health-related features of the NWLDC are shown in table 1. Clinical measures include body mass index (BMI), glycated haemoglobin (HbA1c), systolic and diastolic blood pressures, blood cholesterol (including total, low-density lipoprotein (LDL) and high-density lipoprotein (HDL)) and measures of kidney function (estimated glomerular filtration rate (eGFR)), captured within the 5 years prior to diabetes diagnosis. Baseline values were extracted and are summarised in table 2.

**Table 1:** General characteristics of T2DM, T1DM, GDM and other diabetes types

Characteristics	T2DM	T1DM	GDM	Others
n (%)	279 067 (79.7)	17 638 (5)	33 590 (9.6)	6916 (1.9)
Available demographic information, n	274 502	17 062	33 525	6710
N after filtering ambiguous dates cases	271 343	16 908	32 637	6401
Follow-up years since diabetes diagnosis				
Median duration (IQR)	9.0 (4.1–15.8)	12.7 (5.3–21.8)	4.2 (1.6–8.5)	4.0 (1.5–9.0)
Mortality				
Number of deaths, n (%)	56 521 (20.8)	2883 (17.0)	206 (<1.0)	2639 (41.0)
Median years since diagnosis (IQR)	11.7 (5.7–18.9)	15.6 (6.1–26.3)	15.5 (8.2–25.8)	4.4 (1.6–9.1)
Sex, n (%)				
Male	151 660 (55.8)	9662 (57.1)	–	3644 (56.9)
Female	119 679 (44.1)	7245 (42.8)	32 637	2757 (43.0)
Missing or unknown	5 (*<*1.0)	1 (*<*1.0)	–	–
Age at diabetes diagnosis (years)				
Median (IQR)	56.0 (46.0–66.0)	25.0 (13.0–40.0)	33.0 (29.0–37.0)	60.0 (45.0–72.0)
Of unknown, ambiguous cases excluded	589	–	866	159
Ethnicity, n (%)				
South Asian	86 967 (32.0)	2230 (13.1)	10 558 (32.3)	1307 (20.2)
White	83 842 (30.8)	9465 (55.9)	8352 (25.5)	1870 (28.9)
Other	50 051 (18.4)	1800 (10.6)	8081 (24.7)	755 (11.7)
Black	32 262 (11.8)	1652 (9.8)	3373 (10.3)	603 (9.3)
Unknown	8933 (3.2)	1060 (6.3)	694 (2.12)	1728 (26.7)
Mixed	7116 (2.6)	607 (3.5)	1042 (3.18)	135 (2.1)
Chinese	2355 (*<*1.0)	101 (*<*1.0)	570 (1.7)	54 (*<*1.0)
Diabetes age by ethnicity – median (IQR) years				
Black	55 (43–64)	24 (13–42)	34 (30–38)	53 (40–64)
Chinese	58 (47–67)	24 (16–37)	35 (32–38)	53 (34.5–67)
Mixed	55 (44–64)	20 (11–35)	34 (30–38)	51 (37.5–68.5)
Other	52 (42–62)	24 (12–39)	34 (30–38)	51 (38–65)
South Asian	53 (41–62)	29 (15–45)	33 (30–37)	54 (40–67)
Unknown	60 (46–71)	36 (19–62)	33 (29–37)	68 (57–77)
White	61 (49–71)	23 (13–37)	35 (31–38)	62 (47–75)
Deprivation Index IMD, n (%)				
IMD 1 – IMD 2 (most deprived)	45 907 (17.2)	2886 (17.8)	5550 (17.3)	1178 (18.1)
IMD 3 – IMD 4	61 199 (23.0)	3310 (20.5)	7147 (22.3)	1515 (23.4)
IMD 5 – IMD 6	72 817 (27.3)	4027 (24.8)	8616 (26.9)	1621 (25)
IMD 7 – IMD 8	56 228 (21.1)	3673 (22.6)	6965 (21.7)	1414 (21.8)
IMD 9 – IMD 10 (least deprived)	29 686 (11.1)	2271 (14)	3702 (11.5)	47 (11.5)
N of missing, unknown cases	8366	877	1408	235
Smoking status, n (%)				
People who have never smoked	136 998 (50.4)	3927 (23.2)	22 741 (70)	2634 (41.1)
People who smoke lightly	58 986 (21.7)	3761 (22.2)	4033 (12.4)	1134 (17.7)
People who smoke moderately	15 932 (5.9)	874 (5.2)	683 (2.1)	286 (4.5)
People who smoke heavily	8343 (3.1)	377 (2.2)	220 (<1)	160 (2.5)
People who smoke generally	79 213 (29.2)	5335 (31.6)	5768 (17.7)	1692 (26.4)

GDM, gestational diabetes mellitus; IMD, Index of Multiple Deprivation; T1DM, type 1 diabetes mellitus.

**Table 2:** Average baseline clinical parameters measured within 5 years prior to diabetes diagnosis, stratified by T2DM, T1DM, GDM and other diabetes types

Measurements within 5 years before diabetes diagnosis	T2DM	T1DM	GDM	Others
BMI (kg/m²)				
Number	189 971	5560	23 285	3670
Median (IQR)	29.5 (26.2–33.6)	24.1 (20.7–27.9)	26.0 (22.8–30.1)	27.9 (24.4–32.3)
BMI <18.5, underweight (N, %)	964 (*<*1.0)	698 (12.5)	578 (2.4)	73 (1.9)
BMI 18.5–25, normal (N, %)	30 154 (15.8)	2413 (43.41	9305 (38.7)	989 (26.9)
BMI 25–30, overweight (N, %)	69 999 (36.8)	1499 (26.9)	7916 (3.0)	1256 (34.2)
BMI 30–40, obese (N, %)	74 895 (39.4)	834 (15.0)	5552 (23.1)	1168 (31.8)
BMI ≥40, severely obese (N, %)	13 959 (7.3)	115 (2.0)	637 (2.6)	184 (5.0)
Systolic blood pressure (mm Hg)				
Number	212 327	5573	26 509	4282
Median (IQR)	135.4 (126.4–145.0)	125.6 (116.0–137.1)	115.0 (108.6–122.0)	134.5 (124.0–145.6)
Diastolic blood pressure (mm Hg)				
Number	212 298	5570	26 510	4281
Median (IQR)	81.7 (76.6–87.6)	77.8 (70.5–83.5)	73.6 (69.0–79.0)	80.0 (74.4–86.2)
HbA1c (mmol/mol)				
Number	135 276	1880	11 354	2482
Median (IQR)	49 (46.2–55.0)	52.8 (43.1–71.5)	37.0 (34.0–40.0)	47.0 (42.5–53.5)
Total cholesterol level (mmol/L)				
Number	192 426	3223	11 194	3240
Median (IQR)	5.1 (4.4–5.8)	5 (4.3–5.7)	4.7 (4.1–5.3)	4.96 (4.25–5.73)
Total number of cholesterol <5	85 941	1565	7017	1637
Total number of cholesterol *≥*5	106 485	1658	4177	1603
LDL (mmol/L)				
Number	161 994	2354	10 892	3084
Median (IQR)	3 (2.4–3.6)	2.9 (2.3–3.5)	1.3 (1.1–1.5)	1.2 (1.0–1.4)
HDL (mmol/L)				
Number	161 994	2885	10 892	3084
Median (IQR)	1.1 (1.0–1.3)	1.2 (1.0–1.5)	1.3 (1.1–1.5)	1.2 (1.0–1.4)
eGFR (mL/min/1.73 m^2^)				
Number	12 752	170	1036	382
Median (IQR)	90.0 (78.3–90.0)	90.0 (89.8–90.0)	90.0 (90.0–90.0)	90.00 (80.28–90.00)

BMI, body mass index; eGFR, estimated glomerular filtration rate; GDM, gestational diabetes mellitus; HbA1c, glycated haemoglobin; HDL, high-density lipoprotein; LDL, low-density lipoprotein; T1DM, type 1 diabetes mellitus.

Quantitative variables were handled as follows: BMI was categorised using standard clinical cut-points (<18.5, 18.5–24.9, 25–29.9, 30–39.9, ≥40 kg/m²) to align with WHO classifications and facilitate clinical interpretation of weight status. Total cholesterol was dichotomised at 5 mmol/L, a commonly used threshold in cardiovascular risk assessment. All other continuous variables (systolic and diastolic blood pressure, HbA1c, LDL, HDL, and eGFR) were retained as continuous measures in statistical models.

The incidence rates for common complications after diabetes diagnosis computed and are detailed in table 3. Microvascular complications demonstrated the highest cumulative incidence, with diabetic retinopathy being the highest with 74.6 cases per 1000 person-years, followed by hypertension at 51.0 (formally, both a microvascular and macrovascular complication), and kidney disease at 31.4. Other complications such as dementia and coronary heart disease were also associated with intermediate levels of incidence (ie, 7.1 and 10.4 cases per 1000 person-years, respectively).

**Table 3:** Prevalent cases, at-risk patient populations, incident cases and incidence rates of major comorbidities among patients with T2DM per 1000 person-years in ascending order of incidence

Category	Prevalent cases	Patients at risk (incident-free population)	Incident cases	Person-years	Incidence rate per 1000 person-years
Retinopathy	4339	267 784	137 833	1 845 328	74.69
Hypertension	102 163	169 960	66 638	1 308 921	50.91
Kidney diseases	11 347	260 776	81 641	2 597 231	31.43
Coronary heart disease	22 589	249 534	26 910	2 599 368	10.35
Depression	29 050	243 073	18 819	2 630 936	7.15
Anxiety disorders	22 700	249 423	16 571	2 735 915	6.06
Heart failure	4708	267 415	16 519	2 943 257	5.61
Strokes	6074	266 049	13 584	2 923 438	4.65
Dementia	2092	270 031	13 384	2 983 334	4.49
Peripheral artery disease	2604	269 519	10 002	2 964 886	3.37
Foot ulcer	1923	270 200	8503	2 980 682	2.85
Transient ischaemic attack	3129	268 994	6144	2 980 196	2.06
Liver diseases	1446	270 677	3001	3 021 405	0.99
Amputation (major and minor)	503	271 620	2847	3 028 423	0.94

T2DM, type 2 diabetes mellitus.

**Figure 2:**
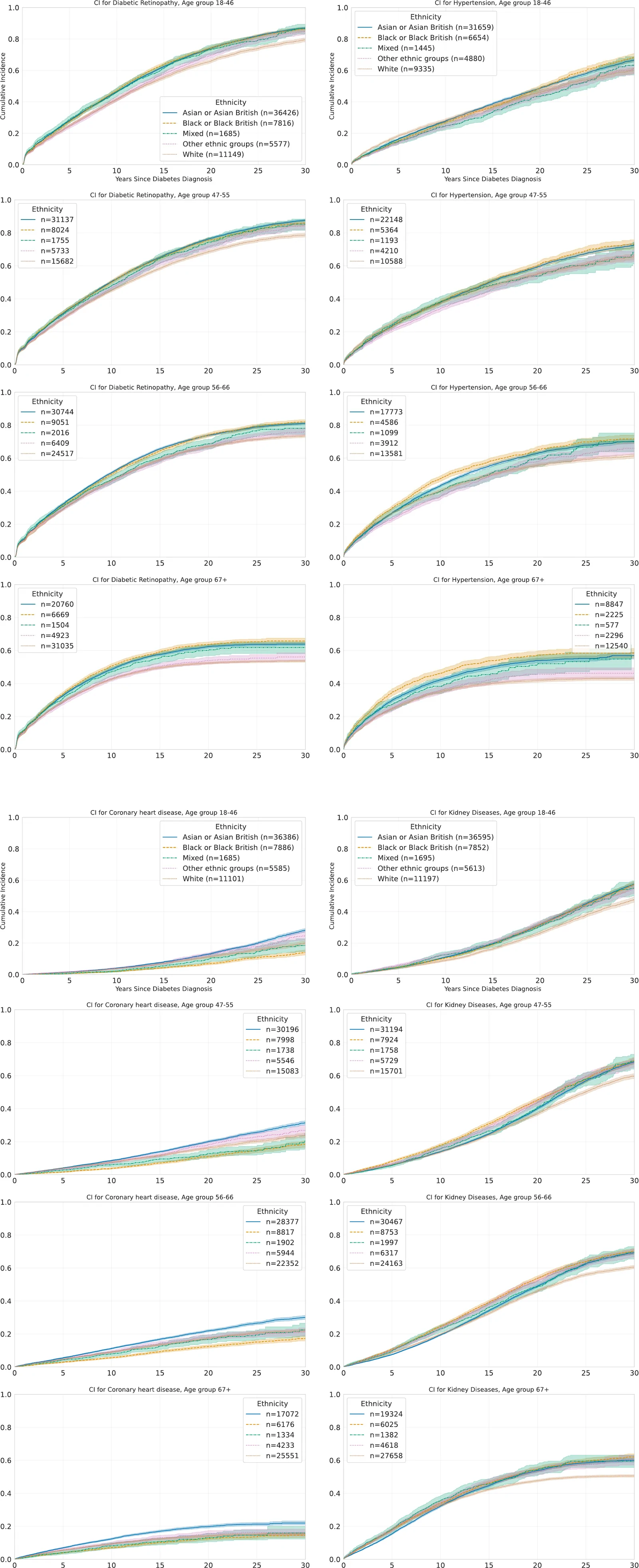
Cumulative incidence function for different diabetes complications, stratified by age and ethnicity.

Analytical methods included cumulative incidence function (CIF) stratified by age and ethnicity, using the Aalen-Johansen estimator, as shown in figure 2 for diabetic retinopathy, hypertension, kidney diseases and coronary heart disease, the four complications with the highest incidence rates per 1000 person-years. CIF curves for the remaining complications by age group are provided in online supplemental figures S4–S6. We used the non-parametric Aalen-Johansen model to estimate the probability of developing a complication, while accounting for mortality as a competing risk. This approach considers death as a competing event that may preclude the event of interest from occurring, and if not considered, it would otherwise bias probability estimates upward. To consider age-related differences in disease progression, CIF were computed across four age groups defined by sample quartiles (18–46, 47–55, 56–66, >67) to ensure balanced sample sizes across age strata. Multivariable linear regression models examined demographic effects on BMI and HbA1c (online supplemental figures S7 and S8). Time-varying Cox models assessed risk factors for diabetic retinopathy, hypertension, kidney disease and stroke, controlling for age, sex, ethnicity and deprivation. The log HRs for these analyses are shown in online supplemental figure S9.

## Findings to date

Several important findings emerged. First, diabetic retinopathy was the most common and temporally occurring complication, with the highest incidence. This is unsurprising as diabetic retinopathy has a central role in how the WHO and other expert bodies set blood glucose thresholds for diagnosing diabetes. The current WHO diagnostic criteria for diabetes on glucose tolerance testing is a fasting plasma glucose of ≥7.0 mmol/L (126 mg/dL) or a 2-hour plasma glucose of ≥11.1 mmol/L (200 mg/dL).^13^ Hypertension occurred frequently both before and after diagnosis, underlining its dual role as risk factor and complication. Kidney disease largely occurred after diabetes diagnosis.

Mental health conditions were also prevalent, with depression and anxiety occurring across the entire timeline, indicating ongoing psychosocial distress. Amputations and foot ulcers largely occurred post-diagnosis, with onset typically delayed by 5–15 years.

In terms of sociodemographic, ethnicity emerged as a powerful determinant of risk of diabetes-related complications. Regression analyses showed modest but significant effects of ethnicity, age, sex and deprivation on BMI and HbA1c. For example, Asians had significantly higher pre-diabetes HbA1c than whites, with Asian men showing the highest overall levels. Younger patients also showed higher HbA1c prior to diagnosis.

Time-varying Cox models revealed clustering between cardiovascular and renal complications. Hypertension, kidney disease and heart failure were strongly interconnected, confirming the concept of a cardiometabolic renal syndrome.^14 15^ Age gradients were clear, with patients over 58 at highest risk of all health outcomes. Ethnic disparities were persistent, with black, Asian, mixed and other groups at elevated risk compared with white patients.^16^ Socioeconomic inequities were evident, with deprivation associated with higher risks.

These findings are consistent with prior evidence that minority and disadvantaged groups bear disproportionate burdens of complications.^16 17^ They highlight the need for identifying at-risk individuals earlier and designing targeted interventions to delay or prevent diabetes complications.

## Strengths and limitations

The major strength of this cohort is its size, diversity and integration. Covering 337 2711 patients with diabetes, with linked data across primary, secondary, mental health and social care, it represents one of the largest multiethnic diabetes cohorts in the UK. The population includes substantial numbers of patients of Asian and black ethnic origin, groups often under-represented in research but disproportionately affected by diabetes. This diversity enhances generalisability and allows examination of ethnic health disparities.

Another strength is the richness of measures. Longitudinal clinical data, laboratory tests, complications and socioeconomic indicators enable multidomain analyses. The linkage of datasets across care sectors provides a holistic view of patient trajectories. The regular update to the system means that future analyses can track ongoing trends, though the current work used a static snapshot for reproducibility.

There are, however, limitations. Misclassification of diabetes type is a persistent challenge, arising from overlaps in coding and the complexity of SNOMED CT. Additionally, the assigned diabetes type may change over time as the disease evolves, and the true diabetes type becomes clearer in an individual. We mitigated this through hierarchical classification rules, but residual misclassification may remain. Similarly, women with GDM who later progress to persistent T2DM retain a GDM classification under our earliest-code convention; complications arising after this progression are attributed to the GDM group rather than T2DM, which may underestimate T2DM-associated complication risk and inflate that of GDM. The absence of medication data in the current analysis limited refinements of classification, although future work will incorporate prescriptions to distinguish T2DM from insulin-treated T1DM cases more reliably.

Another weakness is that routine health records may include missing or inaccurate data, and updates to WSIC can affect reproducibility. While our static snapshot ensured consistency, this necessarily freezes a dynamic dataset. Additionally, although demographic factors explained some variance in BMI and HbA1c, much variation remains unmeasured, likely reflecting genetics, behaviours and environmental exposures.

A further limitation concerns the cumulative incidence (Aalen-Johansen) analyses. Beyond the competing-risk treatment of mortality already accounted for by the estimator, the cumulative incidence analyses also assume independent censoring, that patients lost to follow-up through de-registration or migration out of NWL share the same underlying risk as those who remain under observation. If those lost to follow-up are instead at higher risk, cumulative incidence would be underestimated. We cannot quantify this informative censoring, and incidence estimates should be interpreted accordingly.

## Supplementary material

10.1136/bmjopen-2025-114566 online supplemental figure 1

10.1136/bmjopen-2025-114566 online supplemental figure 2

10.1136/bmjopen-2025-114566 online supplemental figure 3

10.1136/bmjopen-2025-114566 online supplemental file 1

10.1136/bmjopen-2025-114566 online supplemental file 2

10.1136/bmjopen-2025-114566 online supplemental figure 4

10.1136/bmjopen-2025-114566 online supplemental figure 5

10.1136/bmjopen-2025-114566 online supplemental figure 6

10.1136/bmjopen-2025-114566 online supplemental figure 7

10.1136/bmjopen-2025-114566 online supplemental figure 8

10.1136/bmjopen-2025-114566 online supplemental figure 9

## Data Availability

Data may be obtained from a third party and are not publicly available.
