# Supplementary material for: Large-scale multiethnic electronic health record resource for diabetes complications research: the North West London Diabetes Cohort (NWLDC) – cohort profile
Source: BMJ Open. 2026 Sep 4;16(9):e114566. doi: 10.1136/bmjopen-2025-114566 (PMC13548236; doi:10.1136/bmjopen-2025-114566)

Supplementary Materials

Figure S1: Flowchart of numbers included in the NWLDC. The flowchart shows the number of overlaps between the main diabetes categories. Cases with unspecific dates or invalid dates are also excluded in the process. The final number of cases in each category is the population we report on.

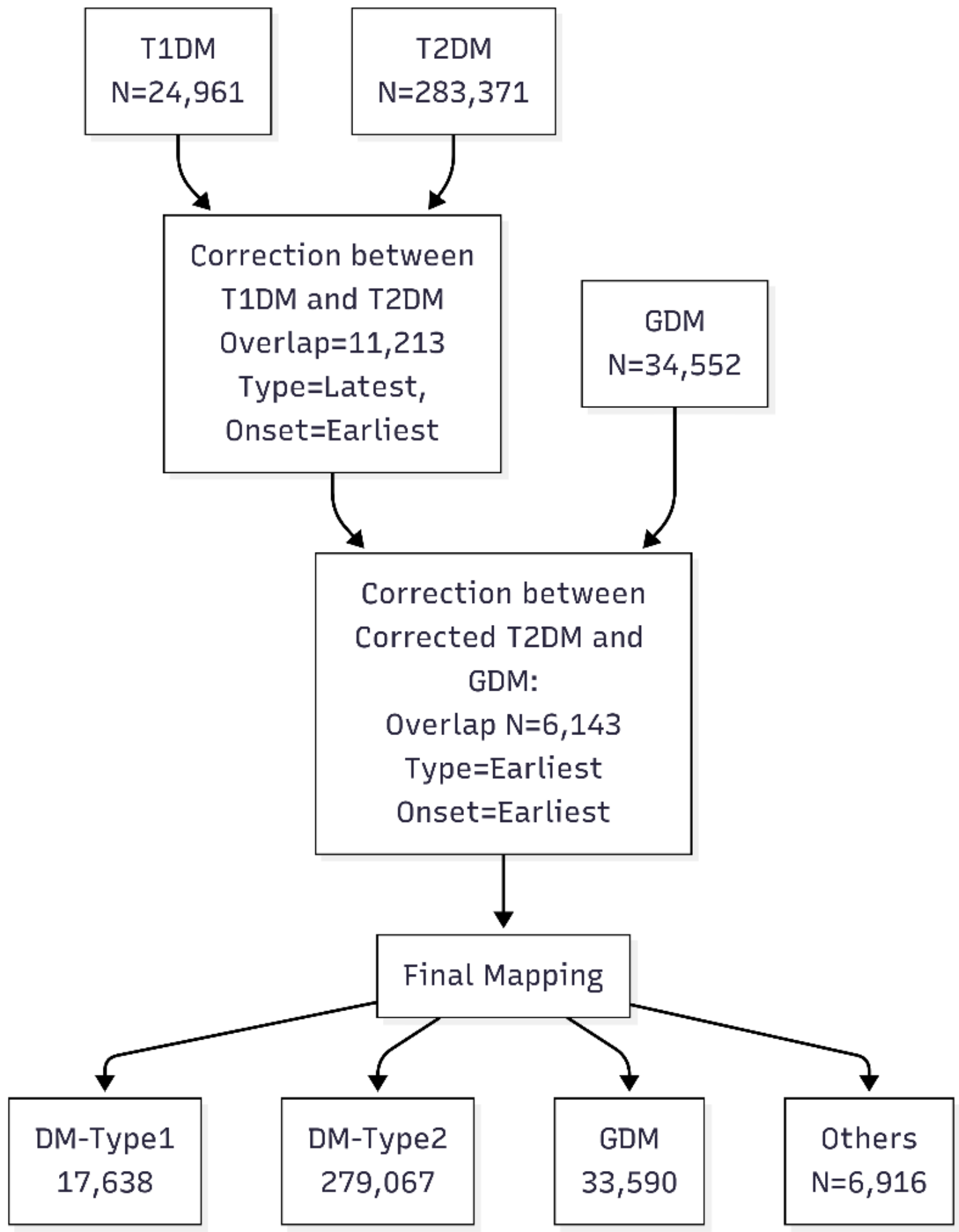

Figure S2: Diabetes diagnosis age distribution for type 1 diabetes (a) before and after (b) correcting for ambiguous cases.

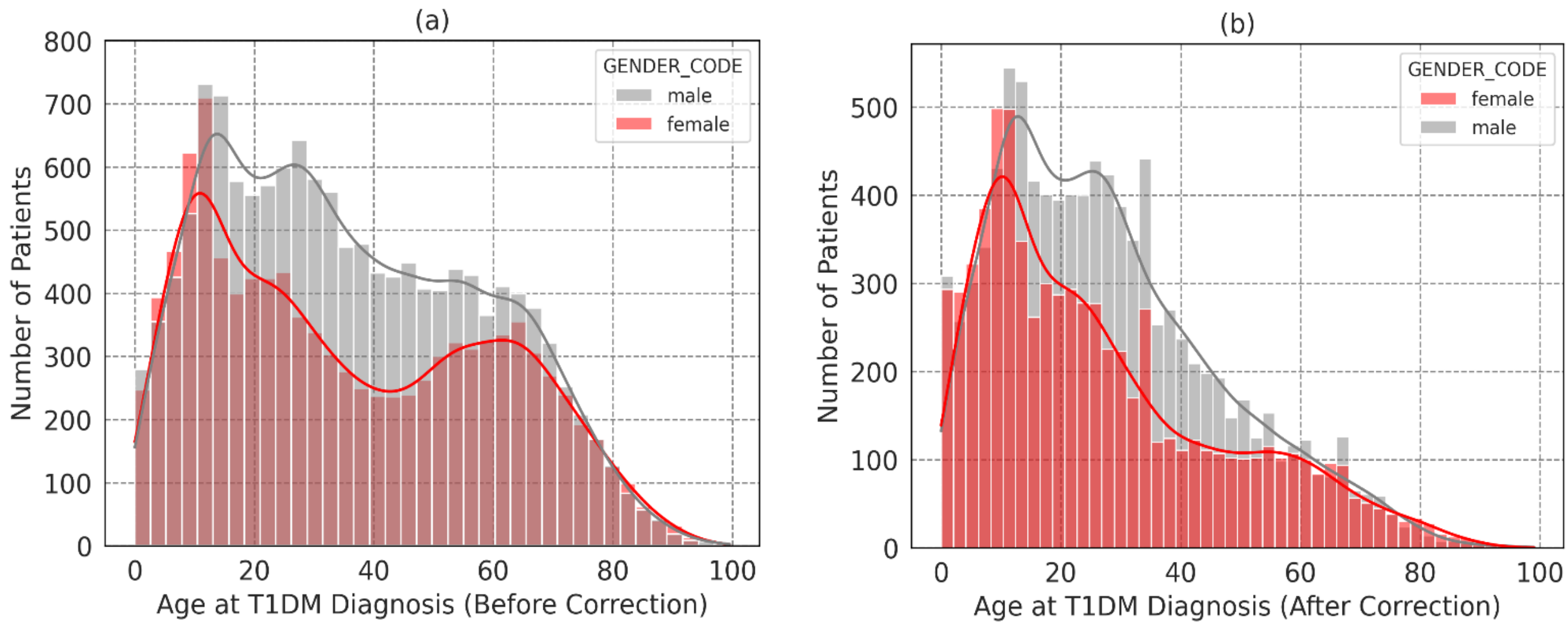

Figure S3: GDM diagnosis: GDM diagnosis age distribution (a) and survival timeline from GDM(b) before/after outlier correction using modified Z-score method.

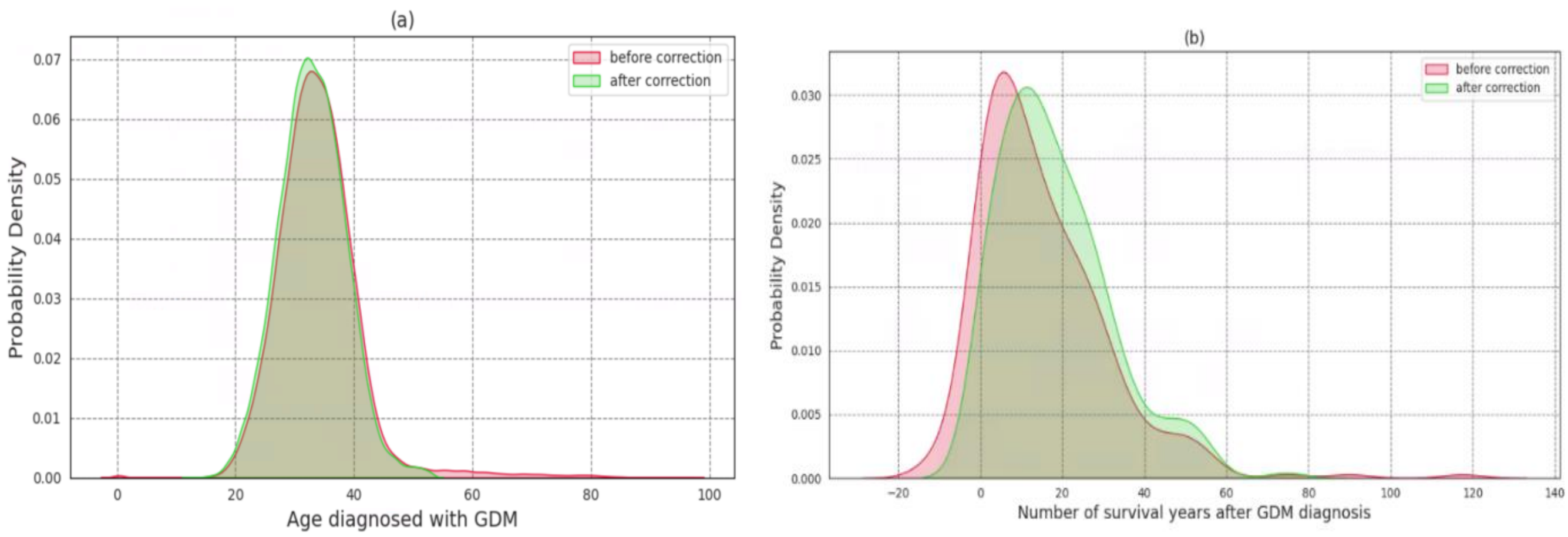

Figure S4: Cumulative incidence of depression, anxiety disorders stratified by ethnicity and quartiles of age at diagnosis (18-46, 47-55, 56-66, and 67+). Curves show the probability of developing each complication over 30 years post-diagnosis.

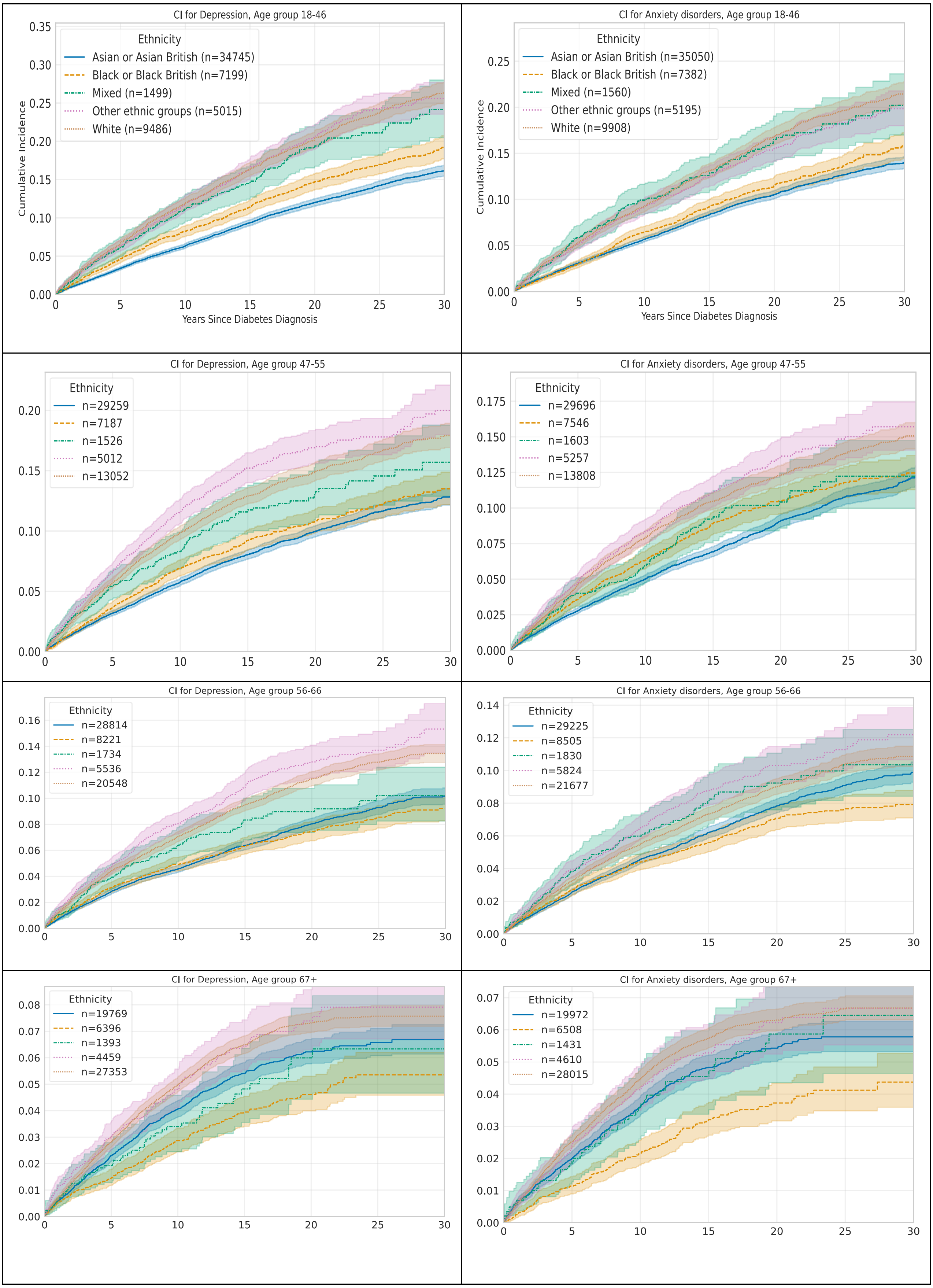
CI for Depression, Age group 18-46
Ethnicity
Asian or Asian British (n=34745)
Black or Black British (n=7199)
Mixed (n=1499)
Other ethnic groups (n=5015)
White (n=9486)
Cumulative Incidence
Years Since Diabetes Diagnosis
CI for Anxiety disorders, Age group 18-46
Ethnicity
Asian or Asian British (n=35050)
Black or Black British (n=7382)
Mixed (n=1560)
Other ethnic groups (n=5195)
White (n=9908)
Cumulative Incidence
Years Since Diabetes Diagnosis
CI for Depression, Age group 47-55
Ethnicity
n=29259
n=7187
n=1526
n=5012
n=13052
CI for Anxiety disorders, Age group 47-55
Ethnicity
n=29696
n=7546
n=1603
n=5257
n=13808
CI for Depression, Age group 56-66
Ethnicity
n=28814
n=8221
n=1734
n=5536
n=20548
CI for Anxiety disorders, Age group 56-66
Ethnicity
n=29225
n=8505
n=1830
n=5824
n=21677
CI for Depression, Age group 67+
Ethnicity
n=19769
n=6396
n=1393
n=4459
n=27353
CI for Anxiety disorders, Age group 67+
Ethnicity
n=19972
n=6508
n=1431
n=4610
n=28015

Figure S5: Cumulative incidence of peripheral artery disease and stroke stratified by ethnicity and quartiles of age at diagnosis (18-46, 47-55, 56-66, and 67+). Curves show the probability of developing each complication over 30 years post-diagnosis.

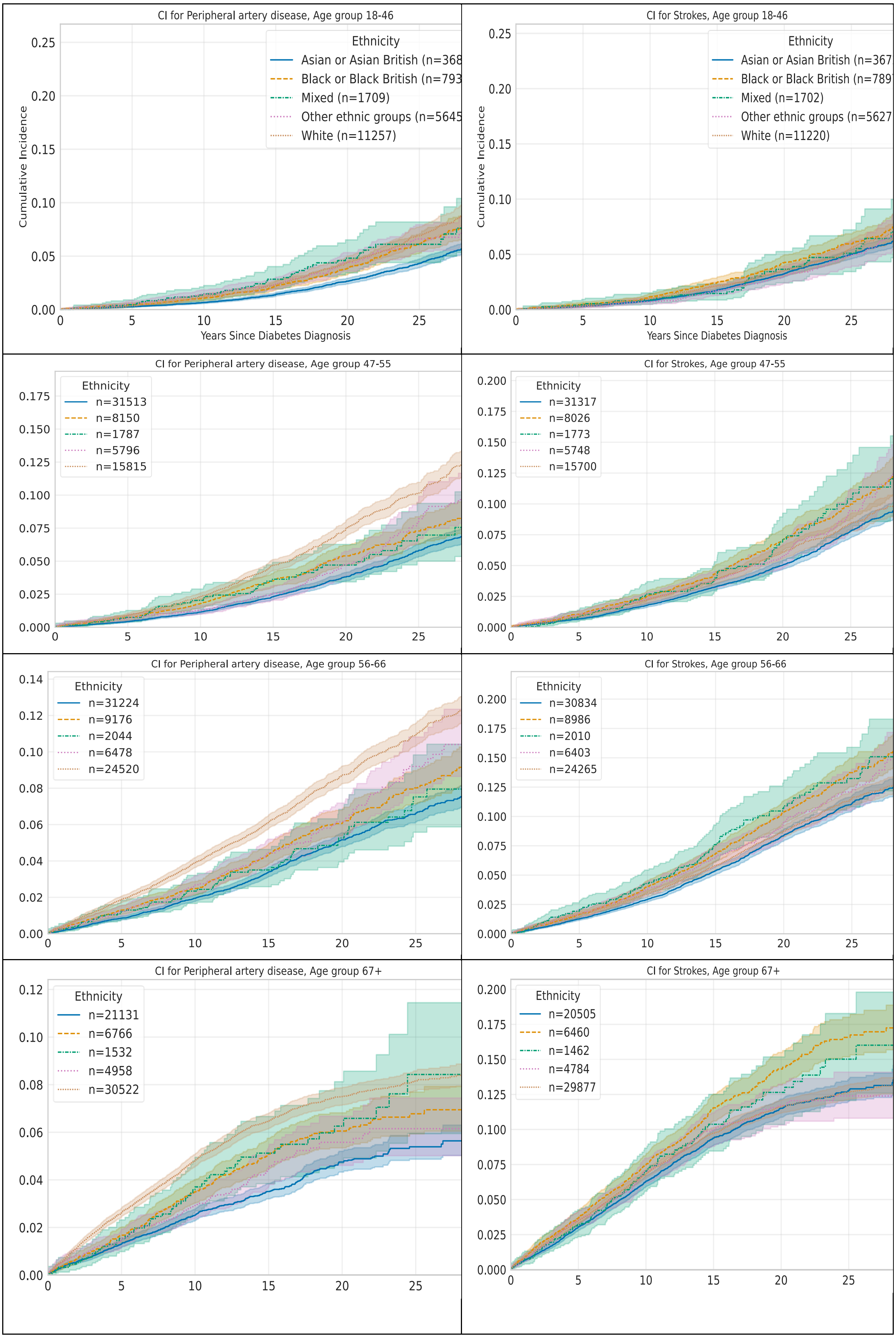
CI for Peripheral artery disease, Age group 18-46
Ethnicity
Asian or Asian British (n=368
Black or Black British (n=793
Mixed (n=1709)
Other ethnic groups (n=5645
White (n=11257)
Cumulative Incidence
Years Since Diabetes Diagnosis
CI for Strokes, Age group 18-46
Ethnicity
Asian or Asian British (n=367
Black or Black British (n=789
Mixed (n=1702)
Other ethnic groups (n=5627
White (n=11220)
Cumulative Incidence
Years Since Diabetes Diagnosis
CI for Peripheral artery disease, Age group 47-55
Ethnicity
n=31513
n=8150
n=1787
n=5796
n=15815
CI for Strokes, Age group 47-55
Ethnicity
n=31317
n=8026
n=1773
n=5748
n=15700
CI for Peripheral artery disease, Age group 56-66
Ethnicity
n=31224
n=9176
n=2044
n=6478
n=24520
CI for Strokes, Age group 56-66
Ethnicity
n=30834
n=8986
n=2010
n=6403
n=24265
CI for Peripheral artery disease, Age group 67+
Ethnicity
n=21131
n=6766
n=1532
n=4958
n=30522
CI for Strokes, Age group 67+
Ethnicity
n=20505
n=6460
n=1462
n=4784
n=29877

Figure S6: Cumulative incidence of heart failure and dementia by ethnicity and quartiles of age at diagnosis (18-46, 47-55, 56-66, and 67+). Curves show the probability of developing each complication over 30 years post-diagnosis.

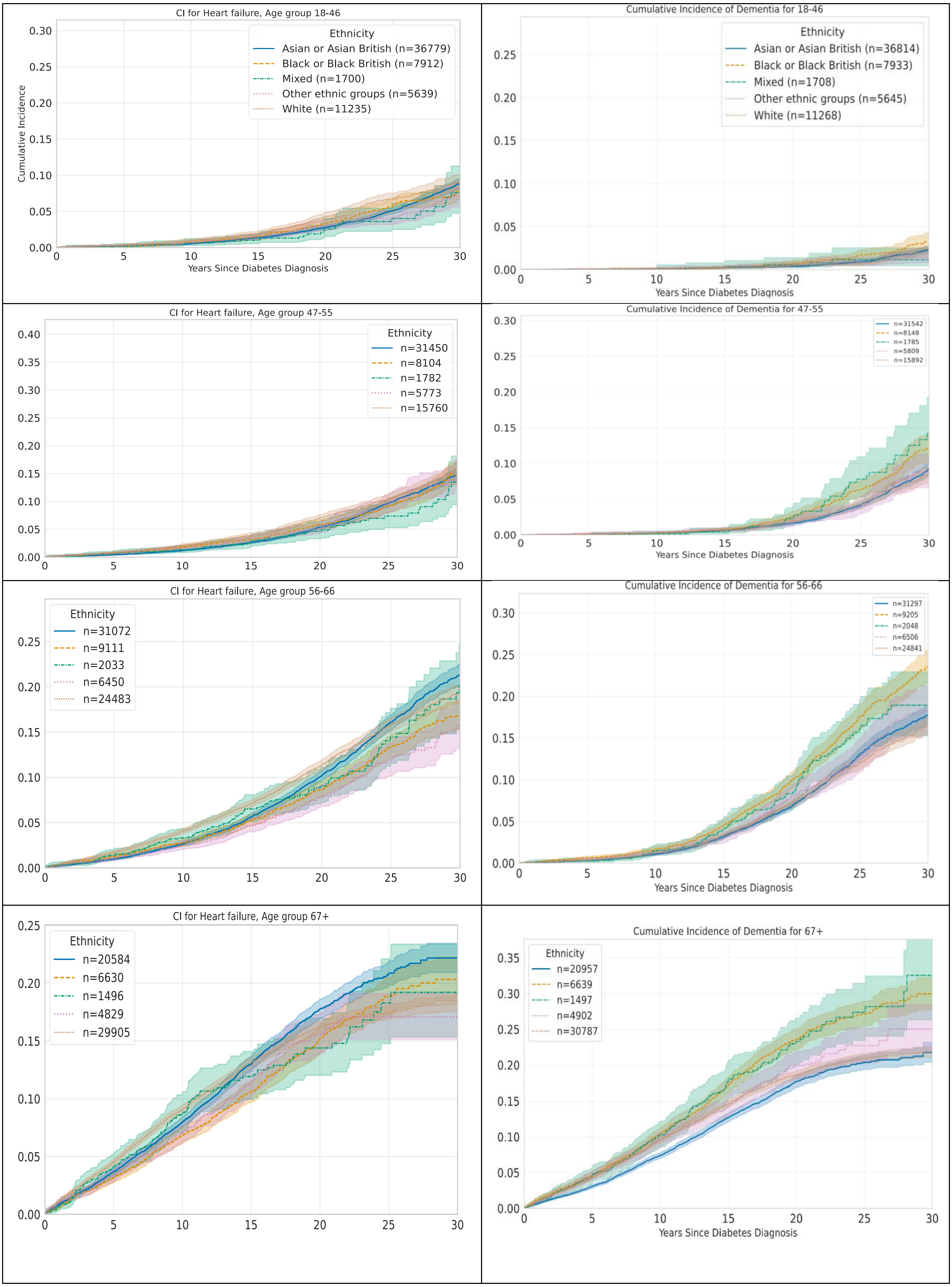
CI for Heart failure, Age group 18-46
Cumulative Incidence
Years Since Diabetes Diagnosis
Ethnicity
Asian or Asian British (n=36779)
Black or Black British (n=7912)
Mixed (n=1700)
Other ethnic groups (n=5639)
White (n=11235)
Cumulative Incidence of Dementia for 18-46
Ethnicity
Asian or Asian British (n=36814)
Black or Black British (n=7933)
Mixed (n=1708)
Other ethnic groups (n=5645)
White (n=11268)
Years Since Diabetes Diagnosis
CI for Heart failure, Age group 47-55
Ethnicity
n=31450
n=8104
n=1782
n=5773
n=15760
Cumulative Incidence of Dementia for 47-55
n=31542
n=8148
n=1785
n=5809
n=15892
Years Since Diabetes Diagnosis
CI for Heart failure, Age group 56-66
Ethnicity
n=31072
n=9111
n=2033
n=6450
n=24483
Cumulative Incidence of Dementia for 56-66
n=31297
n=9205
n=2048
n=6506
n=24841
Years Since Diabetes Diagnosis
CI for Heart failure, Age group 67+
Ethnicity
n=20584
n=6630
n=1496
n=4829
n=29905
Cumulative Incidence of Dementia for 67+
Ethnicity
n=20957
n=6639
n=1497
n=4902
n=30787
Years Since Diabetes Diagnosis

Figure S7: Exploratory Data Analysis Results: (a) Interaction effects between age category and sex on average BMI before diabetes diagnosis, (b) Model diagnostic Q-Q plot for residual normality assessment, and (c) Heatmap showing mean BMI patterns across demographic groups.

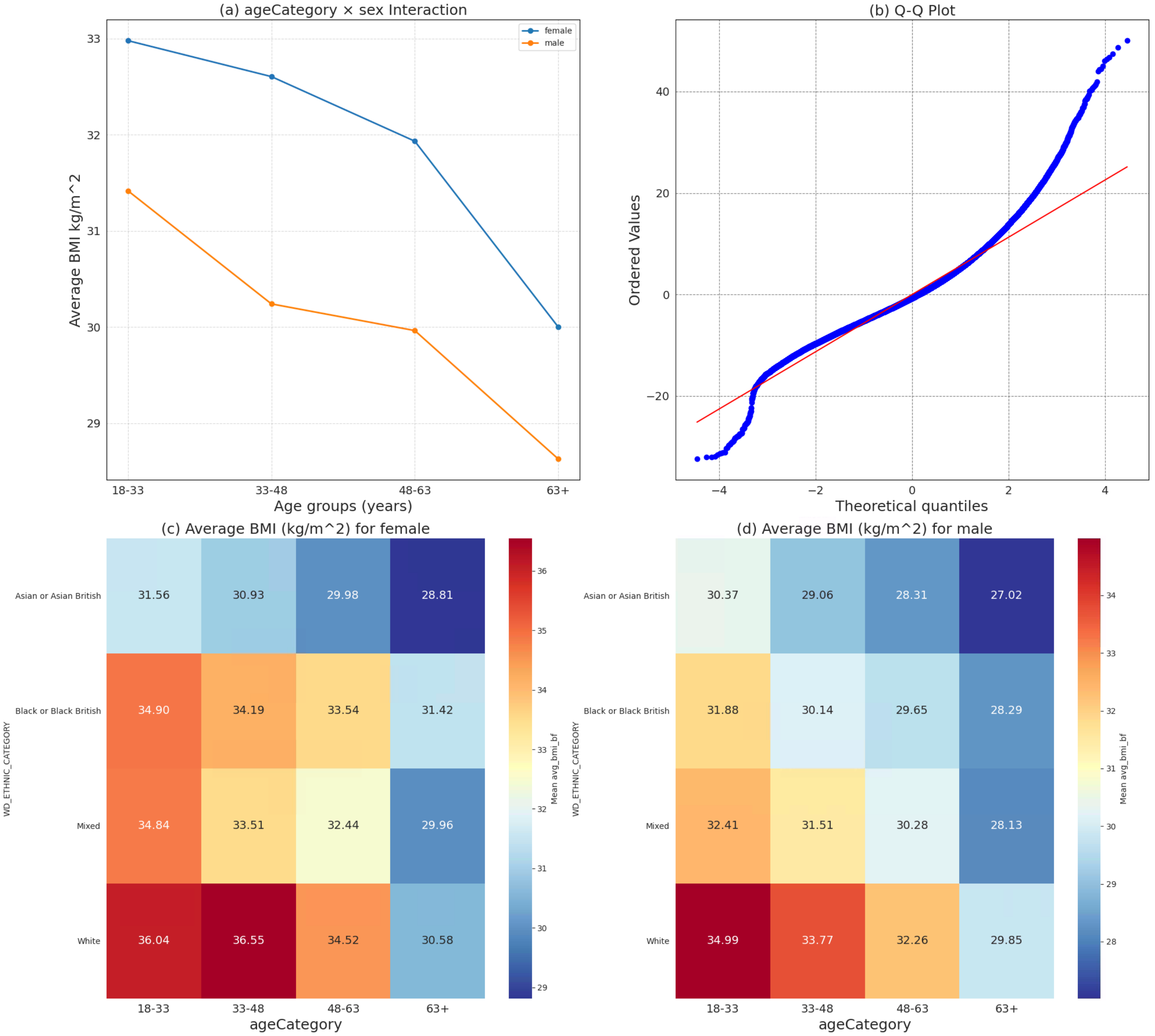

Figure S8: Exploratory data analysis results: (a) interaction effects between age category and sex on average HbA1c before diabetes diagnosis, (b) model diagnostic Q-Q plot for residential normality assessment, and (c) heatmap showing mean BMI patterns across demographic groups.

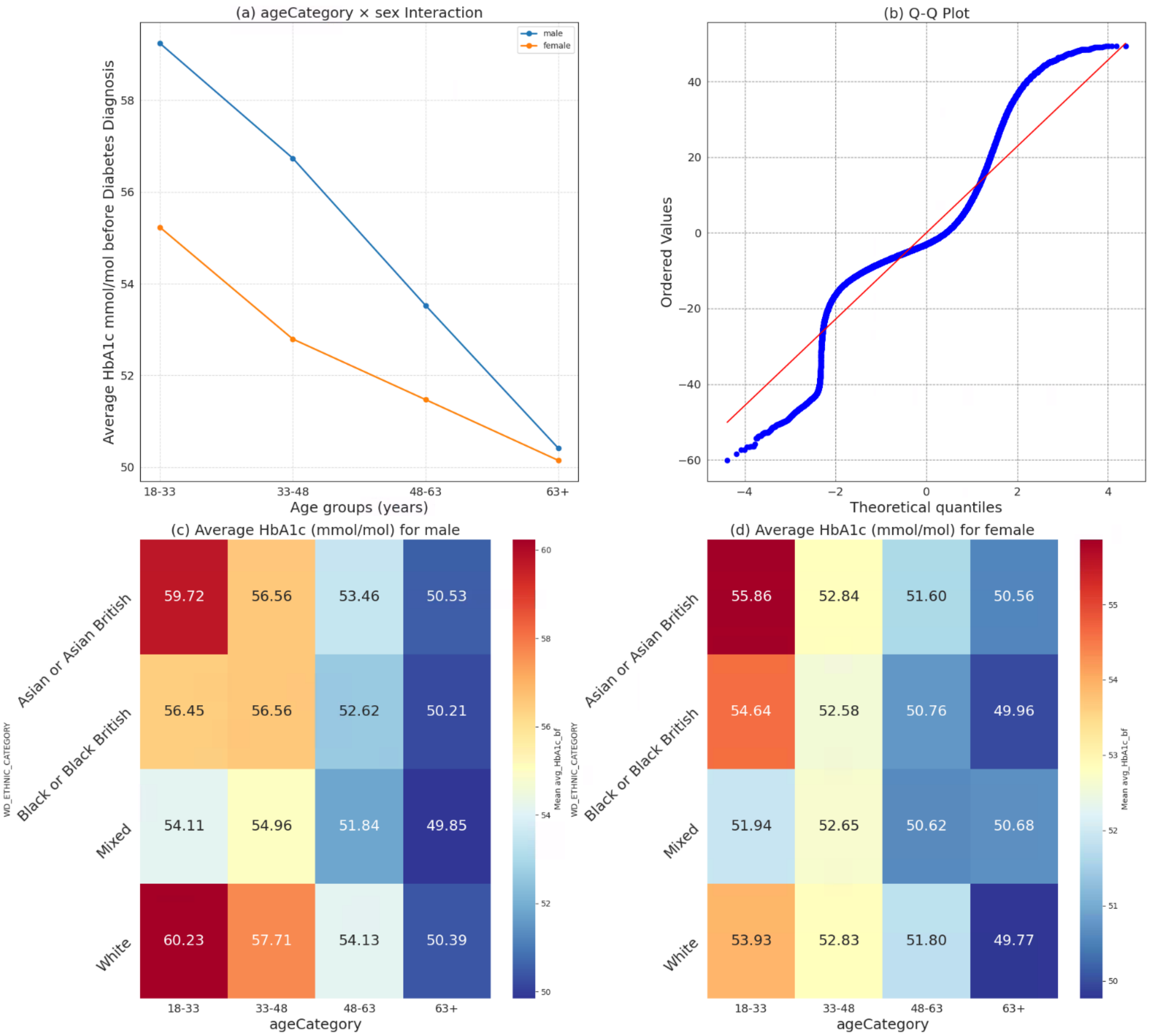

Figure S9 The log hazard ratios (HR) with 95% confidence intervals for developing conditions such as hypertension, kidney disease, diabetic retinopathy and heart diseases. White ethnicity, age group [18-46), and most deprived areas (Deprivation index DI) are used as the reference groups.

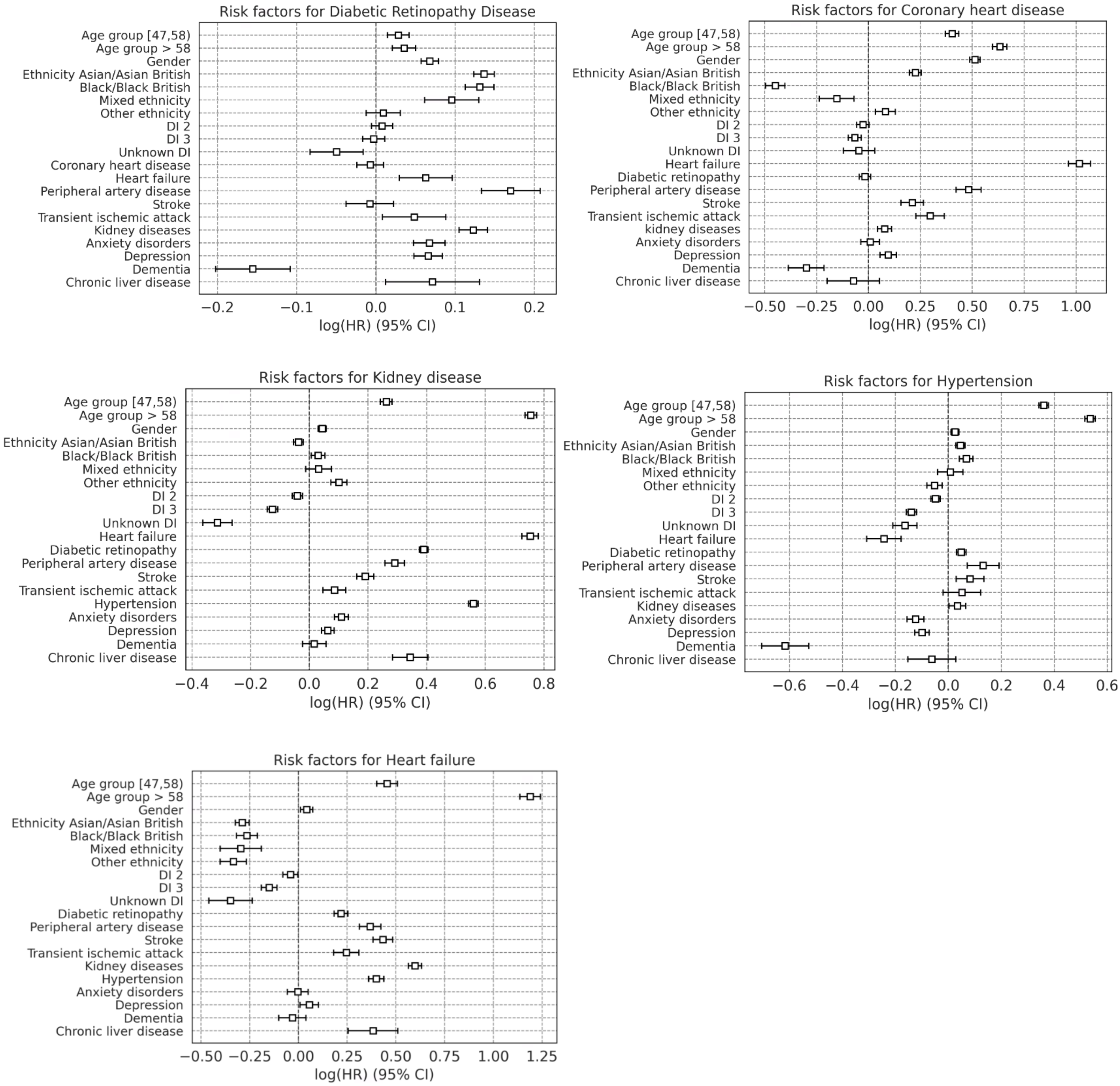

Supplement: Supplementary file 1 [file bmjopen-16-9-s005.pdf]
