# Supplementary material for: Large-scale multiethnic electronic health record resource for diabetes complications research: the North West London Diabetes Cohort (NWLDC) – cohort profile
Source: BMJ Open. 2026 Sep 4;16(9):e114566. doi: 10.1136/bmjopen-2025-114566 (PMC13548236; doi:10.1136/bmjopen-2025-114566)
Supplement: Supplementary file 2 [file bmjopen-2025-114566.reviewer_comments.pdf]

PEER REVIEW HISTORY

BMJ Open publishes all reviews undertaken for accepted manuscripts. Reviewers are asked to complete a checklist review form and are provided with free text boxes to elaborate on their assessment. These free text comments are reproduced below.

ARTICLE DETAILS

Title (Provisional)

Cohort Profile: A Large-Scale Multi-Ethnic Electronic Health Record Resource for Diabetes Complications Research; the North West London Diabetes Cohort (NWLDC)

Authors

Schaefer, Zeinab; Santhirasekaran, Schanhave; Warwick, Alasdair; Grainger, Tabitha; Hingorani, Aroon; Tufail, Adnan; Dixon, Luke; Anderson, John; Moutzouris, Dimitrios; Rudnicka, Alicja R; Roy, Iain Nicholas; Owen, Christopher G.

VERSION 1 - REVIEW

Reviewer 1

Name Mridha, Malay Kanti

Affiliation BRAC James P Grant School of Public Health, BRAC University, Centre for Non-Communicable Diseases and Nutrition

Date 31-Jan-2026

COI None

A nice study with important data. Please find some comments to further improve the clarity of the manuscript.

Abstract: Future plan: This cohort can also be important to report the effectiveness of lifestyle modification practices as a standalone management strategy or in combination with medication for control of blood sugar level and avoidance of complication. The authors need to include this objective in the future plan.

Cohort description: Study population: Please mention how diabetes was classified if a pregnant women with GDM develop type 2 diabetes later on.

Cohort description: What has been measured?: Please include operational definition of 14 major complications, cut-offs for anthropometric and clinical measures and whether there were any changes in the use of these cut-off over time. For example, BP 130/80 mm of Hg cut-off needs to be used for categorizing hypertension among the diabetics.

Findings to date: Specify kidney diseases. Please mention the independent censoring assumption as a limitation as limitation of Aalen–Johansen estimator for cumulative incidence function because lost to follow-up patients can be at higher risk.

Table 1: Use n for sub-groups instead of 'N'. Add a non-smoker category under smoking status.

Table 3: Please reorganize the complication categories in alphabetic order or in descending order based on incidence rate.

VERSION 1 - AUTHOR RESPONSE

Reviewer: 1
Prof. Malay Kanti Mridha, BRAC James P Grant School of Public Health, BRAC University
Comments to the Author:

A nice study with important data. Please find some comments to further improve the clarity of the manuscript.
Abstract:

Future plan: This cohort can also be important to report the effectiveness of lifestyle modification practices as a standalone management strategy or in combination with medication for control of blood sugar level and avoidance of complication. The authors need to include this objective in the future plan.

We thank the reviewer for this comment. We acknowledge the importance of lifestyle modification such as physical activity, dietary changes and weight management. Unfortunately, these metrics are not well recorded in this EHR dataset, as lifestyle factors are inconsistently coded in routine primary care records.

Changes to the paper (Page 2, lines 74-76)

Future Plans

This cohort will be used as a platform for developing and validating prognostic models for diabetes complications, enabling risk stratification and targeted interventions. Future work will incorporate medication data to refine diabetes type classification, examine the effectiveness of anti-diabetic medications in preventing different complications, and address demographic differences in prognostic model performance and prediction accuracy. To better characterise lifestyle, further interrogation of EHR data will examine recording of advice given, including dietary advice, referral to weight management schemes, and presence of alcohol consumption codes.

Cohort description: Study population: Please mention how diabetes was classified if a pregnant woman with GDM develop type 2 diabetes later.

We thank the reviewer for highlighting this. We have clarified that GDM/T2DM overlaps retain the earliest recorded type: a woman coded with GDM who later receives a T2DM code remains classified as GDM (this is mentioned on Page 5, lines 178-180). GDM is a distinct entity defined by detection in pregnancy, and because each patient's index date is anchored on the earliest diabetes code with complications timed relative to it, keeping the earliest type keeps diabetes type and onset date consistent. We acknowledge this convention may under classify complication risk attributable to progression from GDM to T2DM, and we note this as a limitation on page 8, lines 300–303.

Changes to paper: page 8, lines 300–303.

Cohort description: What has been measured? Please include operational definition of 14 major complications, cut-offs for anthropometric and clinical measures and whether there were any changes in the use of these cut-off over time. For example, BP 130/these cut-offsut-off needs to be used for categorizing hypertension among the diabetics.

The 14 complications are identified from SNOMED CT diagnostic codes (provided in full as supplementary material) rather than from clinical measurement thresholds. Because they are ascertained from clinician-entered diagnostic codes, their identification is unaffected by changes in diagnostic cut-offs over time, for example, evolving blood-pressure thresholds for hypertension. The anthropometric and clinical cut-offs applied elsewhere (BMI bands and the total-cholesterol threshold of 5 mmol/L) are used only for descriptive categorisation in Table 2, not for complication extraction. We have clarified this in the Cohort Description (page 5, lines 189–192).

Changes to paper: page 5, lines 189–192.

Findings to date: Specify kidney diseases. Please mention the independent censoring assumption as a limitation as limitation of Aalen–Johansen estimator for cumulative incidence function because lost to follow-up patients can be at higher risk.

We thank the reviewer for the suggestion. We have specified that the kidney disease outcome is a composite comprising CKD stages 1–2, CKD stages 3–5, diabetic nephropathy, and renal dialysis, each identified from curated SNOMED CT cluster codelists. This is now stated in the Cohort Description (page 5 , lines 198-199), and the full code lists are provided as supplementary material.

We agree about the remark regarding the limitation section. The Aalen–Johansen estimator assumes censoring is independent of the complication process, so patients lost to follow-up are assumed to share the same risk as those who remain under observation. This may not hold, as patients who de-register or move out of North West London can differ in risk, and if they are at higher risk the cumulative incidence is underestimated. We have added this as a limitation in the Strengths and Limitations section (page 8, lines 310-315) and Page 3, lines 96-97. We clarified that it is separate from the competing-risk treatment of mortality already accounted for by the estimator.

Table 1: Use n for sub-groups instead of 'N'. Add a non-smoker category under smoking status.

Thank you for these suggestions. We have replaced 'N' with 'n' for all sub-group labels in Table 1, and added a 'Non-smoker' category to the smoking status variable (Table 1, page 14).

Table 3: Please reorganize the complication categories in alphabetic order or in descending order based on incidence rate.

We have modified the table where complications are in descending order based on incidence rate in Table 3 on page 16.
